# Effects of Platelet-Rich Fibrin on Bone Healing Around Implants Placed in Maxillary Sinuses: A Histomorphometric Assessment in Rabbits

**DOI:** 10.3390/jfb15120375

**Published:** 2024-12-12

**Authors:** Gustavo Augusto Grossi-Oliveira, Eduardo Dallazen, Thabet Asbi, João Matheus Fonseca-Santos, Paulo Domingos Ribeiro-Júnior, Jamil A. Shibli, Cinthya Massari Grecco, Osvaldo Magro-Filho, Carlos Fernando Mourão, Doron Haim, Yaniv Mayer, Leonardo P. Faverani

**Affiliations:** 1 Department of Diagnosis and Surgery, Division of Oral and Maxillofacial Surgery and Implantology, School of Dentistry, São Paulo State University (UNESP), Araçatuba 16015-050, SP, Brazil; 2 Maccabi-Dent Research Department, Tel-Aviv 6801298, Israel; 3 Department of Periodontology, Rambam Health Care Campus, Haifa 3525408, Israel; yaniv.mayer@technion.ac.il; 4 Department of Periodontology, Dental Research Division, Guarulhos University, Guarulhos 07023-070, SP, Brazil; 5 Division of Oral and Maxillofacial Surgery and Implantology, School of Dentistry, UNISAGRADO, Bauru 17011-160, SP, Brazil; 6 Department of Basic and Clinical Translational Sciences, School of Dental Medicine, Tufts University, Boston, MA 02111, USA; 7 Department of Oral Diagnosis, Division of Oral and Maxillofacial Surgery, Piracicaba Dental School, University of Campinas (UNICAMP), Piracicaba 13414-903, SP, Brazil; faverani@unicamp.br

**Keywords:** alveolar bone loss, bone substitutes, platelet-rich fibrin, dental implants

## Abstract

This study investigated the effect of platelet-rich fibrin (PRF) on bone healing around implants placed in elevated sinus cavities. Forty New Zealand albino rabbits were divided into eight groups, based on the time of sacrifice (14 or 40 days) and the material used: blood clot (control), hydroxyapatite (HA) from bovine bone, HA combined with PRF, and PRF alone. Each group consisted of five animals (n = 5). A histological analysis measured bone-to-implant contact (BIC) and bone area fraction occupancy (BAFO). The results showed significant increases in the BIC and BAFO values at 40 days compared to 14 days in most groups. At day 14, the HA+PRF group had higher BIC than the clot and the PRF alone groups. At 40 days, HA+PRF maintained the highest BIC across all groups (*p* < 0.05), though it did not show an advantage for BAFO. These findings indicate that combining HA with PRF promotes better osseointegration around implants placed immediately in maxillary sinus augmentation. Given the limited research on PRF’s biological impact, these results underscore the importance of evaluating PRF’s role in peri-implant healing and its potential benefits for clinical use in sinus augmentation.

## 1. Introduction

Alveolar bone resorption and sinus pneumatization in the posterior maxillary regions may prevent the placement of dental implants due to the insufficient height of the sinus floor [1]. Lateral maxillary sinus augmentation is a procedure in which a bone window is created to elevate the sinus membrane, providing a space from the bone margin. This allows for new bone formation, implant placement, and future prosthetic rehabilitation [2,3]. While performing a sinus lift with simultaneous implant placement is acceptable, at least 4–5 mm of residual bone is required for implant stability [4,5]. In such cases, it is crucial to maintain the elevated sinus mucosa in its new position, as membrane collapse around the apex or body of the implant can hinder bone formation and osseointegration [5,6].

Various materials can be used to fill the space underneath the elevated mucosa and create a healthy environment for new bone formation around the implant. Autologous bone grafts are regarded as the gold standard due to their osteogenic, osteoinductive, and osteoconductive properties [7], However, they exhibit higher rates of bone remodeling, resulting in lower bone gain compared to other bone substitutes [5]. To simplify the surgical techniques, dispense additional surgical areas, and reduce the surgical time, numerous bone substitutes have been used, many being hydroxyapatite derived from the bovine bone cortex. These materials show a low resorption rate compared to autologous grafts [8]. However, these biomaterials do not have osteoinductive features, and some local or systemic conditions may delay or impair bone regeneration. Therefore, the use of platelet concentrates, which act as regenerative inducers, has been proposed to enhance and accelerate bone repair [9,10,11]. Choukroun et al. (2006) [12] introduced a second-generation platelet-rich concentrate, known as platelet-rich fibrin (PRF), which is characterized by an autologous fibrin matrix enriched with platelets and leukocytes. PRF serves as a system for the slow release of growth factors, promoting accelerated bone repair. These biochemical components are well known for their synergistic effects on healing processes. For instance, fibronectin plays a crucial role as a guide for cell proliferation and migration [12].

Experimental animal models are essential for understanding the biological response in these procedures, as the analysis of peri-implant bone healing in clinical studies, particularly regarding biomaterials and newly formed calcified tissues, is limited. Rabbits serve as a valuable model for evaluating sinus lift procedures due to the similarity of their anatomical environment to that of humans [13].

Hence, this study aimed to explore the effect of platelet-rich fibrin on bone healing around implants concomitantly placed in elevated sinus cavities.

## 2. Materials and Methods

This study was designed according to the Animal Research: Reporting of In Vivo Experiments—ARRIVE guidelines 2.0 [14]. The research protocol was then approved by the Animal Ethical Committee of the University of Sagrado Coração, School of Dentistry, Bauru, São Paulo, Brazil (#18/14).

A sample size calculation was performed using the SigmaPlot 12.0 program (Exakt Graphs and Data Analysis, San Jose, CA, USA). Using BIC as the primary outcome and drawing from previous studies with similar outcomes and methodologies, the sample size was determined considering a mean difference of 2.8, a standard deviation of 1.7, and a statistical power of 95%. It was calculated that nine samples per time point would ensure statistical homogeneity. However, considering the potential for sample loss during the experiment, five samples were assigned to each group instead of three. In total, 40 animals were included in the study.

The studies included forty albino New Zealand rabbits, approximately 5–6 months old weighing 3–4 kg. The animals were randomly assigned to four groups at each time point using an envelope and lottery system, with both maxillary sinuses filled according to the designated group material. Each group consisted of five animals (n = 5). The animals were sacrificed at either 14 or 40 days. The materials used to fill the sinuses were as follows: Group 1 (Clot group) was filled with a blood clot, Group 2 with hydroxyapatite (HA group) derived from bovine bone cortex (Lumina Bone—Criteria ©, Industry and Trade of Medicinal and Dental Products, São Carlos, Brazil), Group 3 with a combination of HA and PRF (HA+PRF group), and Group 4 with PRF only (PRF group).

The animals underwent an 8 h fasting period prior to surgery. The surgical sites were shaved and then disinfected using 10% polyvinylpyrrolidone iodine (PVP-I; Riodeine^®^, Rioquímica, São José do Rio Preto, Brazil). Anesthesia was induced with ketamine hydrochloride (1%; Vetaset^®^, Fort Dodge Saúde Animal LTDA, Campinas, São Paulo, Brazil) administered at a dose of 10 mg/kg, in combination with xylazine hydrochloride (2%; Dopaser^®^, Laborat Calier do Brasil Ltd., São Paulo, Brazil) at 5 mg/kg. Additionally, the midline of the nasal dorsum was locally infiltrated with 2% mepivacaine containing epinephrine (1:100,000; Mepiadre 100^®^, DFL LTDA, Rio de Janeiro, Brazil) at a volume of 0.3 mL/kg.

A midline incision was performed along the nasal dorsum. Following flap elevation, the nasal bone was bilaterally exposed up to the nasofrontal sutures. Bilateral circular bony windows, each with a diameter of 5 mm, were delineated using a trephine drill (Neodent^®^, Curitiba, Brazil). The centers of these windows were positioned approximately 5–6 mm lateral to the midline of the nasal dorsum and 5–6 mm anterior to the apex of the nasofrontal suture. The osteotomy was completed using a No. 1011 round drill (KG Sorensen^®^, Cotia, Brazil), reducing the diameter of the bony window to 2.5–3 mm. Once the sinus mucosa was exposed, sinus floor elevation instruments (Neodent^®^, Curitiba, Brazil) were utilized to carefully detach the mucosa along the medial and lateral margins, as well as approximately 3–4 mm along the mesial and distal aspects of the osteotomy.

Venous blood was drawn from the right or left auricular vein using a 30 × 8 mm needle inserted into the central or marginal vessel. A 5 mL syringe was used to collect 5 cm^3^ of venous blood. After collection, the blood was placed in a sterile stainless-steel vat for 10 min to allow coagulation, and the resulting clot was used to fill the maxillary sinus in the control group (clot group). For PRF processing, the same blood collection procedure was followed. The collected blood was processed according to the protocol presented by Choukroun in 2006 to obtain PRF. The blood was placed in a plastic tube without anticoagulant and centrifuged at 3000 rotations per minute (approximately 400 g in the maximum relative centrifugal force) for 10 min. After centrifugation, three distinct layers were visible in the tube: the upper containing platelet-poor plasma, the intermediate PRF layer, and the lower layer of red blood corpuscle. The intermediate PRF layer was used to fill the maxillary sinuses for the PRF groups (HA+PRF and PRF groups).

**Figure 1:**
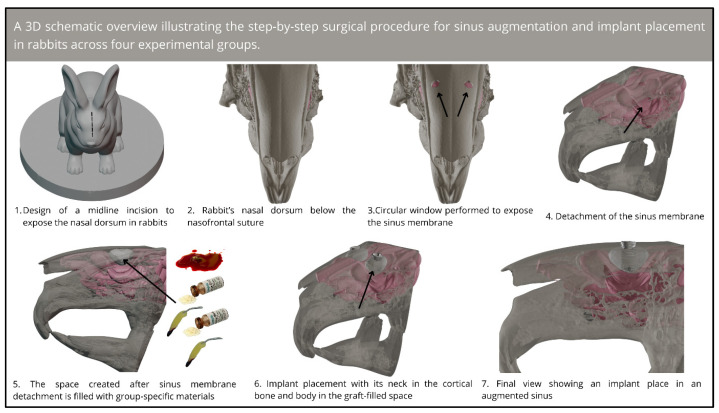
Three-dimensional schematic overview of the step-by-step surgical procedure for sinus augmentation and implant placement in rabbits.

Subsequently, the space which was obtained after sinus mucosa elevation was filled using biomaterials according to each group (the clot, HA, HA+PRF, or PRF groups). The volume of material, clot, or platelet-rich fibrin (PRF) placed into the sinus was not standardized, as it was determined based on the anatomical capacity of each sinus and the clinical judgment necessary to achieve complete filling. Nevertheless, consistency was maintained by meticulously monitoring and documenting the volume utilized in each case. This procedure was followed by the placement of a titanium implant (Implalife^®^—Indústria de produtos médicos odontológicos, Jales, Brazil), with the diameter of 3.6 mm and length of 6.5 mm. The surface of the implant was double-acid-etched, with the exclusion of the coronal portion (approximately 1 mm), which was turned. The base of the neck was placed at the level of the cortical bone (in equicrestal position), and the entire body of the implant was placed inside the bone tissue and the sinus. A cover screw was placed, and the flaps were sutured using resorbable polyglactin 910 (Vicryl 5-0^®^, Ethicon, Johnson & Johnson, São José dos Campos, Brazil) for deep sutures and nylon (Ethilon 5-0^®^, Ethicon, Johnson & Johnson, São José dos Campos, Brazil) for the skin sutures. An illustrative schematic overview of the surgical steps is shown in Figure 1.

Postoperatively, the animals received a single intramuscular dose of antibiotics (Pentabiotico^®^, 0.1 mL/kg; Fort Dodge Saúde Animal Ltd., São Paulo, Brazil) and analgesics (Dipyrone Sodium^®^, 1 mg/kg/day for 3 days; Ariston Indústrias Químicas e Farmacêuticas Ltd.a, São Paulo, Brazil). They were housed individually in labeled cages indicating their experimental group, within a climate-controlled environment at the university’s field laboratory. The animals had ad libitum access to water and a nutritionally balanced diet (Ração Procoelho, Primor^®^, Jaguare, Brazil). Postoperative monitoring included daily inspection of the surgical sites for any clinical signs of complications. Animals presenting with signs of infection at the surgical site or other organs were excluded from further analysis. For euthanasia, sedation was initially achieved using ketamine hydrochloride (1%; Vetaset^®^, Fort Dodge Saúde Animal Ltd., Campinas, São Paulo, Brazil) at a dose of 10 mg/kg in combination with xylazine hydrochloride (2%; Dopaser^®^, Laborat Calier do Brasil Ltd., São Paulo, Brazil) at 5 mg/kg. Euthanasia was performed via intracardiac injection of 150 mL of 0.9% saline solution (Darrow, Rio de Janeiro, Brazil) over 10 min, followed by 1800 mL of 4% formaldehyde for tissue fixation.

The maxilla with the nasal sinus compartments was excised, and block sections of the experimental regions were prepared and maintained in 4% formaldehyde solution (Reagentes Analíticos, Dinâmica Odonto-Hospitalar Ltd., Catanduva, Brazil).

Individual blocks containing the implant and surrounding tissues were fixed in 4% formaldehyde solution, followed by dehydration through a graded ethanol series. The blocks were then embedded in resin (LR White^®^, hard grade; London Resin Company Ltd., Berkshire, UK). To prepare for analysis, the blocks were sectioned through the midline of the implants in the coronal plane using a diamond band saw mounted on a precision slicing machine (Exakt^®^, Apparatebau, Norderstedt, Germany). The sections were subsequently ground to a thickness of approximately 50 µm using a cutting-grinding device (Exakt^®^, Apparatebau, Norderstedt, Germany).

Given that the coronal portion of the implant resides in native bone, and significant differences were anticipated in the apical region (minimal bone formation in the clot group), the analysis focused on the central region of the implant. From each block, one or two histological sections were prepared from the central portion of the implant. These sections were stained with Stevenel’s blue and alizarin red and examined under a standard light microscope for histometric analysis. Histometric evaluations were performed using Eclipse Ci (Nikon Corporation, Tokyo, Japan), equipped with a digital video camera (Digital Sight DS-2Mv, Nikon Corporation, Tokyo, Japan) connected to a computer. The acquired digital images were analyzed by a single calibrated blinded examiner (E.D) for both experimental groups and periods using ImageJ^®^ version 3.1 (U.S. National Institutes of Health, Bethesda, MD, USA). Images at 25× magnification were used to calculate the bone-to-implant contact (BIC; primary outcome) in three threads in the center region of the implant. Images at 25× magnification were used to calculate the bone area fraction occupancy (BAFO; secondary outcome) at the center thread of the implant.

SigmaPlot software version 12.0 was used for statistical analysis. A normality test (Shapiro–Wilk) was performed, followed by parametric tests. All data were analyzed by two-way analysis of variance followed by the Holm–Sidak method with *p* < 0.05, and a confidence interval of 95%, assessing the correlation between BIC and BAFO values, and two periods of analysis (14 and 40 days).

## 3. Results

None of the 40 albino New Zealand rabbits had postoperative complications, and all were included in the BIC and BAFO analyses and descriptive histology.

### 3.1. Descriptive Histology (n = 40)

**Figure 2:**
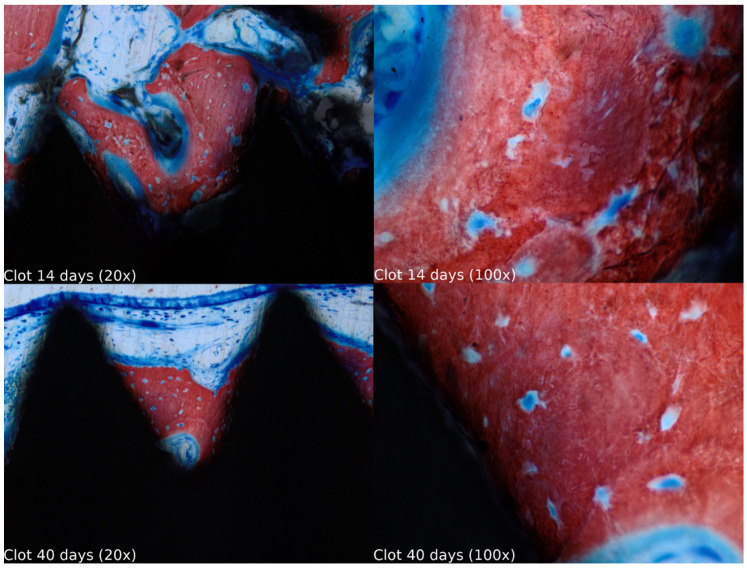
Healing of the clot group at 14 and 40 days at 20× and 100× magnification stained with Stevenel’s blue and alizarin red.

**Figure 3:**
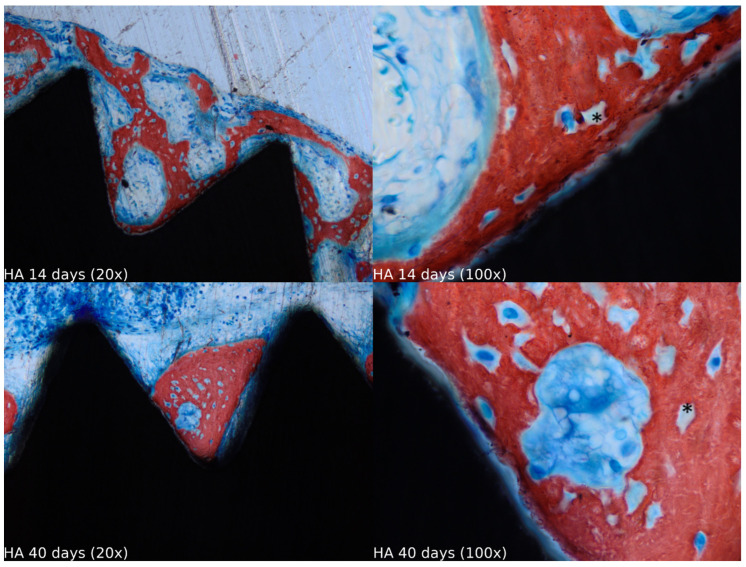
Healing of the HA group at 14 and 40 days at 20× and 100× magnification stained with Stevenel’s blue and alizarin red. * Residual HA particles.

**Figure 4:**
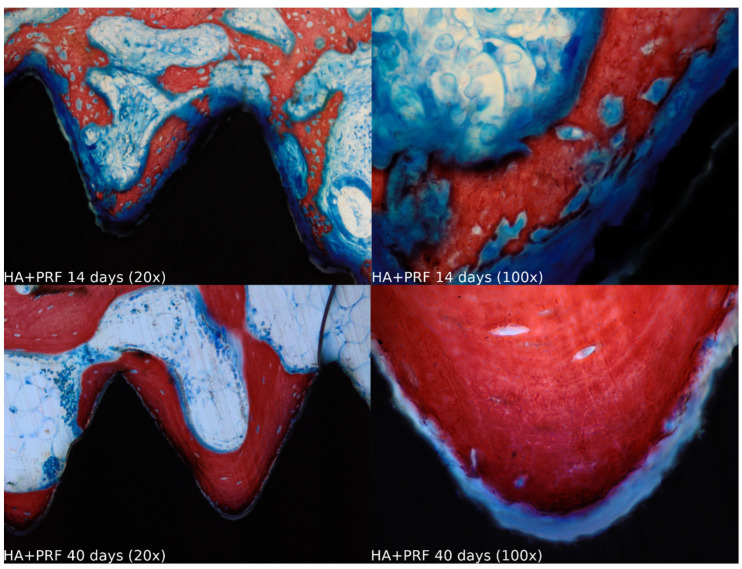
Healing of the HA+PRF group at 14 and 40 days at 20× and 100× magnification stained with Stevenel’s blue and alizarin red.

**Figure 5:**
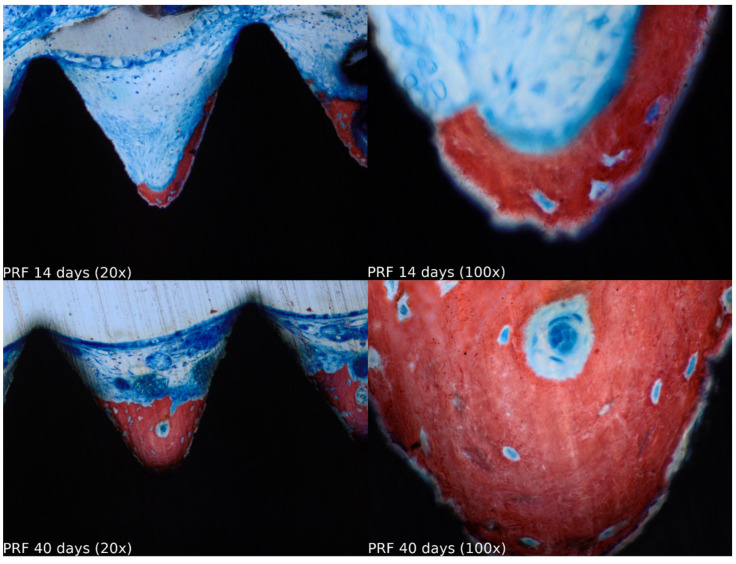
Healing of the PRF group at 14 and 40 days at 20× and 100× magnification stained with Stevenel’s blue and alizarin red.

Biopsies showed similar compositions of histological structures in all groups, without signs of exacerbating inflammatory reactions. At 14 days of healing, the clot, HA, and HA+PRF groups showed similar new bone formation and soft tissue distribution. Only the PRF group showed poor new bone formation and more soft tissue close to the implant threads. At 40 days, the distribution of new bone formation was more homogeneous and showed a higher portion of bone tissue in all groups. The histological analysis showed dynamic healing from 14 to 40 days and revealed increased bone tissue organization, particularly in the PRF groups, which demonstrated lamellar bone formation by 40 days postoperatively. In the HA group, residual HA particles were observed up to the final evaluation period, whereas in the HA+PRF group, no HA particles were present at 40 days, but a decrease in the number of osteocytes and blood vessels was noticed (Figure 2, Figure 3, Figure 4 and Figure 5).

### 3.2. BIC from 14 to 40 Days of Healing (n = 40)

**Figure 6:**
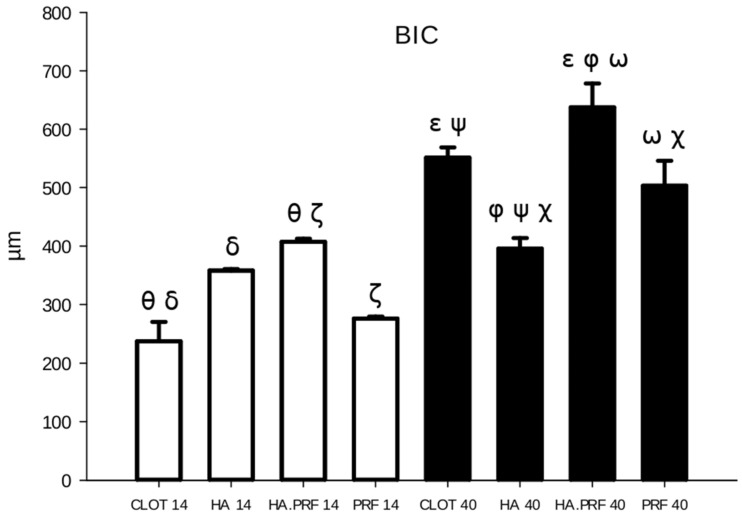
Column graph showing bone-to-implant contact quantity in the experimental groups at 14 and 40 days. Ө δ ζ—statistical difference comparing groups at 14 days of healing. ε φ ω ψ χ—statistical difference comparing groups at 40 days of healing. Only HA (group 2) did not show a statistical difference from 14 to 40 days of healing.

A statistically significant difference in BIC was observed at 40 days of healing compared to 14 days for all groups, except group 2 (HA). The largest mean difference occurred in group 1 (clot), with a value of 313.836 μm^2^, followed by group 3 (HA+PRF) at 230.016 μm^2^, group 4 (PRF) at 227.043 μm^2^, and group 2 (HA) at 36.817 μm^2^ (Figure 6).

### 3.3. BIC at 14 Days of Healing (n = 20)

At 14 days of healing, the mean BIC values were as follows: group 1 (clot) showed 237.276 ± 73.467 μm^2^, group 2 (HA) 358.302 ± 5.589 μm^2^, group 3 (HA+PRF) 407.222 ± 11.658 μm^2^, and group 4 (PRF) 276.284 ± 7.078 μm^2^. Group 3 (HA+PRF) demonstrated a statistically significant difference in BIC compared to group 1 (clot), with a difference of 169.946 μm^2^ (*p* < 0.001), and compared to group 4 (PRF), with a difference of 130.938 μm^2^ (*p* = 0.005). Group 2 (HA) also showed a statistically significant difference compared to group 1 (clot), with a difference of 121.026 μm^2^ (*p* = 0.009). No statistically significant differences were found between groups 2 (HA) and 4 (PRF), groups 2 (HA) and 3 (HA+PRF), or groups 1 (clot) and 4 (PRF) (Figure 6).

### 3.4. BIC at 40 Days of Healing (n = 20)

From the evaluation at 40 days of healing, group 1 (clot) showed a mean of 551.112 ± 39.445 μm^2^; group 2 (HA), 395.173 ± 40.959 μm^2^; group 3 (HA+PRF), 637.237 ± 91.359 μm^2^; and group 4 (PRF), 503.326 ± 95.587 μm^2^. There was a statistical difference in BIC between all groups, except between the clot and PRF groups. The highest mean BIC was observed in group 3 (HA+PRF), which showed a statistically significant difference when compared to group 2 (HA), with a difference of 242.065 μm^2^ (*p* < 0.001),; group 4 (PRF), with a difference of 133.911 μm^2^ (*p* = 0.003); and group 1 (clot), with a difference of 86.126 μm^2^ (*p* = 0.047). Both group 1 (clot) and group 4 (PRF) had higher mean values that were statistically significant compared to group 2 (HA), with differences of 155.939 μm^2^ (*p* < 0.001) and 108.153 μm^2^ (*p* = 0.016), respectively (Figure 6).

### 3.5. BAFO from 14 to 40 Days of Healing (n = 40)

**Figure 7:**
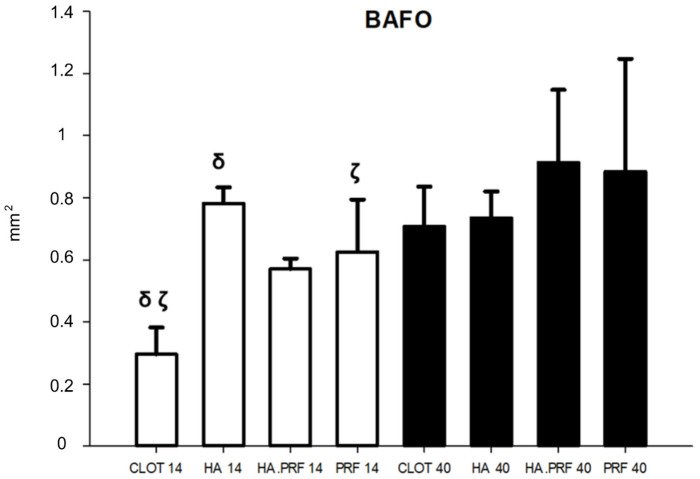
Column graph showing bone area fraction occupancy quantity in the experimental groups at 14 and 40 days. δ ζ—statistical difference comparing groups at 14 days of healing. Only HA (group 2) did not show a statistical difference from 14 to 40 days of healing.

An increase in BAFO was observed, with a statistically significant difference between the values at 40 days compared to 14 days of healing in all groups, except for group 2 (HA). The largest mean difference was found in group 1 (clot), with 0.411 mm^2^ (*p* < 0.01), followed by group 3 (HA+PRF), with 0.342 mm^2^ (*p* = 0.004); group 4 (PRF), with 0.258 mm^2^ (*p* = 0.028); and group 2 (HA), with 0.046 mm^2^ (*p* = 0.682) (Figure 7).

### 3.6. BAFO at 14 Days of Healing (n = 20)

From the evaluation at 14 days of healing, group 1 showed a mean of 0.296 mm^2^ ± 0.086; group 2, 0.782 mm^2^ ± 0.052; group 3, 0.570 mm^2^ ± 0.034; and group 4, 0.626 mm^2^ ± 0.169. Significant differences were observed in the HA, HA+PRF, and PRF groups in comparison to the clot group. However, only the differences between group 2 (HA), with a difference of 0.486 mm^2^, and group 4 (PRF), with a difference of 0.330 mm^2^, represented a statistically significant difference. In contrast, group 3 (HA+PRF) presented a difference of 0.274 mm^2^ compared to group 1 (clot), which did not represent a statistical difference. The other associations also did not show statistical differences (*p* > 0.05) (Figure 7).

### 3.7. BAFO at 40 Days of Healing (n = 20)

From the evaluation at 40 days of healing, group 1 showed a mean of 0.707 mm^2^ ± 0.129; group 2, 0.735 mm^2^ ± 0.085; group 3, 0.913 mm^2^ ± 0.235; and group 4, 0.884 mm^2^ ± 0.363. There was no statistical difference in BAFO among any of the groups (Figure 7).

## 4. Discussion

This animal study aimed to assess the effect of PRF on osteointegration of implants in maxillary sinus augmentation in rabbits. This study showed improved healing when adding PRF to bone substitute in such a procedure.

Interestingly, although hydroxyapatite presents little resorption [15,16], the histological analysis revealed that PRF promoted the remodeling of HA, consistent with findings from a previous study demonstrating increased resorption of the bone substitute when combined with PRF [17]. However, other studies have reported no significant difference in the residual bone substitute when used either alone or in conjunction with PRF [18,19].

In their seminal study, Choukroun et al. (2006) [12] demonstrated that the combination of PRF with freeze-dried bone allograft can significantly reduce bone healing time in sinus lift surgery. They reported a comparable increase in new bone formation percentages two months earlier than when using bone allograft alone. In our study, no statistically significant difference was observed between the use of PRF with HA and HA alone at 14 days of healing. However, by 40 days, the PRF and HA combination clearly yielded the best BIC results, showing a statistically significant difference from all other groups. This is in line with other studies which showed that using PRF may improve osteointegration by presenting higher BIC values [20,21]. These findings suggest that the combination of PRF with HA may enhance BIC in dental implants placed concurrently with sinus lift surgery, likely due to the synergistic effect of HA’s osteoconductive properties and the osteoinductive chemokines within PRF’s fibrin matrix.

Zhang et al. (2012) [22] conducted a clinical study to assess the combination of PRF and deproteinized bovine bone mineral in maxillary sinus augmentation, comparing it to the use of deproteinized bovine bone mineral alone. Six months after the histological evaluation, the authors observed similar composition and distribution in both groups. There were no signs of inflammatory reaction and a homogeneous distribution of biomaterial particles, with new bone formation bridging the gaps between the particles. However, the histomorphometric analysis revealed that the group using PRF in combination with deproteinized bovine bone showed a 1.4-fold increase in new bone formation compared to the group using deproteinized bovine bone alone. In our study, however, no difference was found in the quantity of new bone (BAFO) between the group combining PRF and HA and the group using HA alone. Another study showed similar BAFO values in the PRF and non-PRF groups [20]. This difference may be attributed to using implants with different surface topography.

A meta-analysis of randomized controlled trials conducted by Liu et al. (2019) [11] showed no difference in some analyses between the utilization of PRF and bone graft in maxillary sinus augmentation and the use of bone graft alone. In this analysis, there was no difference in the amount of new bone formation and survival rate of implants.

Nevertheless, even if a maxillary sinus lift is a successful procedure with long-term follow-up, some clinical situations may impair the results. Sinus pathologies and systemic diseases, particularly those related to bone metabolism alterations, such as diabetes, osteoporosis, thyroid, and adrenal dysfunction, may delay healing and increase the risk of infection [23,24,25,26].

Thus, the association of HA+PRF may be feasible for clinical applications and is considered useful, particularly for critical situations, such as in large maxillary sinus, low bone density areas, or cases of sinus membrane perforation, where PRF may cover the perforation and also protect against possible sinus contamination. BAFO showed no difference among groups analyzed in this study at 40 days postoperatively; however, the primary parameter for osseointegration, BIC, showed superiority for HA+PRF in the two experimental periods (14 and 40 days). This result supports the additional effect of PRF associated with particulate biomaterials.

This study has some limitations, common for in vivo studies, such as the accelerated repair rate in rabbits compared to humans; the small maxillary sinus dimensions found in an animal, which can lead to difficulties in the procedure; and also the selection and acquisition of the implant to be used in the study.

Another limitation is the assessment of only the central portion of the implant, excluding the apical region, where differences are expected. Due to technical laboratory constraints, we focused on the central area, which may show minimal differences that are difficult to detect in human studies but more observable in animal models. Lastly, we relied solely on histology to evaluate bone formation; incorporating other methods, such as micro-CT, could provide additional insights into this process.

## 5. Conclusions

Within the limitations of this animal study, it can be concluded that adding PRF to bone substitute improved the osseointegration of implants placed immediately after maxillary sinus elevation in rabbits.

## Data Availability

The original contributions presented in this study are included in the article. Further inquiries can be directed to the corresponding author.
